# Gene Panel Approach to Screen for Hereditary Cerebral Small Vessel Disease: A Proof-of-Concept Study

**DOI:** 10.3390/genes17010098

**Published:** 2026-01-19

**Authors:** Chiara Ferraro, Silvia Giliani, Alessandro Pezzini

**Affiliations:** 1 Neurology Clinic, Department of Medicine and Surgery, University of Parma, 43126 Parma, Italy; chiara.ferraro@unipr.it; 2 Department of Molecular and Translational Medicine, University of Brescia, 25123 Brescia, Italy; silvia.giliani@unibs.it; 3 Department of Medicine and Surgery, University of Parma, 43126 Parma, Italy; 4 Stroke Care Program, Department of Emergency, Parma University Hospital, 43126 Parma, Italy

**Keywords:** hereditary cerebral small vessel disease, monogenic stroke, stroke in young adults

## Abstract

**Background:** The predictive performance of pre-screening phenotype-based algorithms in selecting patients with cerebral small vessel disease (cSVD), one of the main causes of ischaemic and haemorrhagic stroke and dementia, more likely to harbor clinically relevant genetic variants (CRGVs) has to date been poorly defined, making it a clinical challenge to decide which patients to screen for hereditary cSVD (hcSVD). **Methods:** We designed a high-throughput gene panel to identify variants in 27 candidate genes associated with cSVD and screened patients selected by a specific phenotype-based algorithm at one comprehensive stroke center from 2020 to 2023. We categorized participants into two sub-groups defined by pre-screening likelihood of hcSVD (hcSVD; High-Probability Group, HPG vs. Low-Probability Group, LPG) and compared the results of molecular analysis. **Results:** Among 65 probands, we detected four (6.1%) pathogenic CRGVs and seven (10.7%) variants of unknown significance (VUSs) in 11 (16.9%) patients. Pathogenic CRGVs were exclusively detected in the HPG (4/22 probands), corresponding to an 18.2% prevalence of hcSVD in this group. Of the seven VUSs, five (22.7%) were detected in the HPG vs. two (4.6%) in the LPG. **Conclusions:** The pragmatic algorithm we are proposing has the potential to help clinicians in identifying patients who are more likely to harbor monogenic disease.

## 1. Introduction

Cerebral small vessel disease (cSVD), one of the leading causes of ischaemic and haemorrhagic stroke and dementia, is estimated to be related to single-gene disorders (hereditary cSVD, hcSVD) in only approximately 1.5% of cases, according to recent data [1]. Next-generation sequencing (NGS) techniques, including gene panels, whole-exome sequencing (WES), and whole-genome sequencing (WGS), have expanded our knowledge on genetic disorders, including monogenic cSVD, and our capability to identify disease-causing mutations has improved. Notwithstanding, while access to WES/WGS procedures within the clinical context can still be challenging and expensive at many centers, NGS panels might represent an effective and suitable first choice in these settings. A major challenge generated by NGS, however, is how to select patients for specific genetic testing. Because of the substantial overlap in both clinical and neuroimaging phenotypes, it is not always easy to distinguish hcSVD from more common sporadic cSVD counterparts. Furthermore, the hereditary nature of a patient’s cSVD may be masked by a milder phenotype or the absence of extracerebral features that are part of the clinical spectrum of most hcSVDs, by the coexistence of vascular risk factors, and by an uninformative family history [2]. This implies a substantial risk of failing to diagnose hcSVD in some cases.

Based on these premises, we carried out a pilot study in which we tested the predictive performance of a phenotype-based algorithm aimed at increasing the ability of hcSVD diagnosis by selecting individuals more likely affected by a monogenic disease in clinical practice.

## 2. Materials and Methods

### 2.1. Patient Selection

The recruitment period for the present study was August 2020 through March 2023. Written informed consent was obtained for all participants (or next of kin). All consecutive patients whose stroke was likely caused by cSVD were considered potentially eligible for the present study. cSVD was retained as the likely cause of ischemic stroke when the Trial of Org 10,172 in Acute Stroke Treatment (TOAST) criteria and the Causative Classification System for Ischemic Stroke (CSS) criteria [3,4] for cSVD were met. The detection of covert vascular lesions including lacunes, white matter hyperintensities (WMHs) of presumed vascular origin, perivascular spaces (PVSs), and cerebral microbleeds (CMBs) on brain MRI further supported the hypothesis of cSVD as the likely pathogenic mechanism of stroke in these cases [5]. Since several monogenic forms of ischemic stroke also may cause intracerebral hemorrhage (ICH), we also considered cerebral bleeding as a possible cSVD phenotype. In addition to information on demographics, conventional vascular risk factors and stroke characteristics were obtained from data on the history of stroke or dementia among first-degree family members (father, mother, and siblings) via a face-to-face interview with the patient and/or the closest family member(s) and collected neuroimaging features (brain MRI, MR angiography, or CT angiography). Participants were those patients who were suspected to be affected by hcSVD based on a specific Mendelian stroke phenotype-based algorithm, including (1) age at first stroke onset ≤ 55 years, (2) <2 conventional major cardiovascular risk factors (such as hypertension, hypercholesterolaemia, diabetes mellitus, obesity [defined as Body Mass Index (BMI) > 30], and active smoking), (3) history of premature (≤55 years) stroke or dementia in first-degree relatives, (4) specific clinical/radiological features of Mendelian stroke (such as, for example, angiokeratoma or O’Sullivan’s sign [6]). cSVD patients who met any two of the above criteria were included in the group of participants highly suspected to be affected by Mendelian stroke (High-Probability Group, HPG), while those complying with one criterion were included in the group with a low pre-test probability of having a monogenic disease (Low-Probability Group, LPG).

### 2.2. Construction of the Panel

A targeted panel approach was applied including genes known to be involved in cSVD, namely genes that were reported to harbor causal mutations for hcSVD (*n* = 7; Table 1, Group A) or for diseases in which small subcortical infarcts presenting with a lacunar syndrome are part of the clinical phenotype (*n* = 20; Table 1, Group B).

Blood samples were collected from all participants, and DNA was extracted from ethylenediaminetetraacetic acid samples. After the preparation of Ampliseq on demanded amplicons, the NGS panel was run using IonChef automatic libraries preparation on an Ion GeneStudio™ S5 Prime (ThermoFisher Scientific, Waltham, MA, USA). With vertical coverage under 30 reads, samples have been Sanger sequenced for the specific exons. Every variation found using NGS was confirmed using Sanger’s sequencing [7]. The detected variants were reclassified based on the interpretation guidelines from the American College of Medical Genetics and Genomics (ACMG) [8] into clinically relevant genetic variants (CRGVs; pathogenic or likely pathogenic) and variants of uncertain significance (VUSs).

## 3. Results

Overall, of the 349 patients who received a diagnosis of cSVD during the study period, 65 (mean age, 47 ± 7.5 years; males, 40%; ischemic stroke, 90.8%; Supplementary Materials Table S1) qualified for the analysis and consented to participate in this study. Of these, 22 (33.8%) met the criteria for inclusion in the HPG, with the remaining 43 (66.2%) in the LPG. Twenty-seven (41.5%) patients had no conventional vascular risk factors, while seventeen (26.1%) had a parent or sibling who had experienced a stroke. There were no significant differences in demographic and clinical characteristics, as well as in the distribution of major cardiovascular risk factors between the HPG and the LPG.

We identified four (6.1%) pathogenic CRGVs and seven (10.7%) VUSs in 11 (16.9%) patients. None of the patients harboring CRGVs had clinical or imaging characteristics of the corresponding monogenic disease.

Patient no. 1 was a 27-year-old female native to Pakistan affected by cSVD with recurrent ischemic strokes, who had no history of major cardiovascular risk factors other than being a smoker of 10 cigarettes per day for 9 years before hospital admission, and an unremarkable family history. As previously reported [7], she harbored the NM_001282225.2:exon2:c.G139A:p.G47R mutation in the *ADA2*, encoding for adenosine deaminase 2, in the homozygous state, consistent with the diagnosis of adenosine deaminase 2 deficiency (DADA2). Other specific features of DADA2 syndrome were lacking at the time of acute stroke and became clinically obvious only later at follow-up evaluations.

Patient no. 2 was a 37-year-old male hospitalized for acute ischemic stroke due to cSVD, with no risk factors other than a personal history of episodic migraine with visual aura and a history of premature dementia and stroke in first-degree relatives, who harbored the NM_000435:c.C619T:p.R207C mutation in the *NOTCH3*, in the heterozygous state, consistent with the diagnosis of Cerebral Autosomal Dominant Arteriopathy with Subcortical Infarcts and Leukoencephalopathy (CADASIL).

Patient no. 3 was a 43-year-old male, with a personal history of hypertension and recurrent acute ischemic strokes caused by cSVD but no other remarkable clinical features or family history. He harbored the NM_001171.6:c.C3421T:p.R1141Ter mutation in the *ABCC6*, in the heterozygous state, consistent with the diagnosis of Pseudoxanthoma elasticum.

Patient no. 4 was a 47-year-old female with a personal history of recurrent intracerebral hemorrhages lacking major cardiovascular risk factors, with documented early-onset dementia and stroke in first-degree relatives (though it was not possible to differentiate between ischemic strokes versus hemorrhagic strokes). She harbored the NM_000484.4:c.2077G>A:p.Glu693Lys mutation in the *APP*, in a heterozygous state, consistent with the diagnosis of hereditary cerebral amyloid angiopathy.

Pathogenic CRGVs were exclusively detected in the HPG, corresponding to an 18.2% prevalence of hcSVD in this group. Of the seven VUSs, five (22.7%) were detected in the HPG vs. two (4.6%) in the LPG (Table 2).

## 4. Discussion

The main finding of our pilot study was that a rigorous, pragmatic, phenotype-based algorithm, such as the one we tested in the present analysis, emerged as a useful tool for practicing neurologists in the selection of subjects to be screened for hcSVD because of its potential in identifying CRGV carriers. Early age at the time of cSVD onset, a low prevalence (0–1) of major conventional cardiovascular risk factors, and a family history of premature stroke or dementia, when in different combinations, should be viewed as reliable clues for hcSVD, even in the absence of clinical or imaging features suggestive of disease. Based on our algorithm, the likelihood of identifying CRGVs accounted for approximately one in five in the subgroup considered to have a high pre-test probability of carrying a monogenic disease, a finding that provides a rationale for validation in larger patient cohorts [1,9,10]. Obviously, clinicians should bear in mind that the lack of the above red flags is not enough to exclude a priori genetic screening. The possibility of the de novo occurrence of pathogenic variants accounts for the absence of affected family members in some cases, the broad inter-individual variability of the clinical phenotype explains the late age at disease onset in others, and the presence of major vascular risk factors, mainly hypertension, does not exclude a monogenic etiology, especially in cases of suggestive features [11,12]. Furthermore, we are aware of the limitations of our exploratory analysis, which is based on a limited sample of patients and lacks validation on a larger cohort, whose results are not directly comparable with those of other studies conducted to date applying a similar methodological approach but on different populations [1,9,10]. In addition, our data on family history relied solely on participants’ self-reports rather than verified medical records. This potentially introduces recall bias or inaccuracies due to limited familial communication, even though participants were asked to acquire the information from the best possible source. Regarding family history of stroke, we could not differentiate between ischemic strokes versus hemorrhagic strokes or specific ischemic stroke subtypes. Notwithstanding, our findings, consistent with others [9,13], clearly indicate that the pre-test probability of a positive genetic result in these cases is extremely low, such that molecular screening is not always warranted. Also, they emphasize that the application of a targeted cSVD-specific high-throughput sequencing panel, allowing the simultaneous testing of multiple genes underlying a single disease phenotype, might be regarded as a cost-efficient approach in cases of cSVD suspected to be genetically determined.

## Figures and Tables

**Table 1:** Composition of the gene panel.

	Gene	Location	OMIM ID	Inheritance	Associated Mendelian Disorder
Group A	COL4A1	13q34	120130	AD	Angiopathy, hereditary, with nephropathy, aneurysms, and muscle cramps; Brain small vessel disease with or without ocular anomalies; Anterior segment dysgenesis with cerebral involvement; Porencephaly 1; Retinal artery tortuosity
	COL4A2	13q34	120090	AD	Brain small vessel disease 2
	CTSA	20q13.12	613111	AR	Galactosialidosis
	FOXC1	6p25.3	602482	AD	Axenfeld-Rieger syndrome, type 3
	HTRA1	10q26.13	602194	AD, AR	Cerebral autosomal recessive arteriopathy with subcortical infarcts and leukoencephalopathy (CARASIL); Cerebral arteriopathy, autosomal dominant, with subcortical infarcts and leukoencephalopathy, type 2
	NOTCH3	19p13.12	600276	AD	Cerebral arteriopathy with subcortical infarcts and leukoencephalopathy (CADASIL1)
	TREX1	3p21.31	606609	AD	Vasculopathy, retinal, with cerebral leukodystrophy
Group B	ABCC6	16p13.11	603234	AR	Arterial calcification, generalized, of infancy, 2; Pseudoxanthoma elasticum
	ACP5	19p13.2	607944	AR	Spondyloenchondrodysplasia with immune dysregulation
	ACTA2	10q23.31	102620	AD	Moyamoya disease 5; Aortic aneurysm, familial thoracic 6; Multisystemic smooth muscle dysfunction syndrome
	ADAMTS13	9q34.2	274150	AR	Thrombotic thrombocytopenic purpura, hereditary
	APP	21q21.3	104760	AD	Alzheimer disease 1, familial; Cerebral amyloid angiopathy, Dutch, Italian, Iowa, Flemish, Arctic variants
	CBS	21q22.3	613381	AR	Homocystinuria, B6-responsive and nonresponsive types; Thrombosis, hyperhomocysteinemic
	CECR1-ADA2	22q11.1	615688	AR	Vasculitis, autoinflammation, immunodeficiency, and hematologic defects syndrome
	CYP11B1	8q24.3	202010	AR	Adrenal hyperplasia, congenital, due to 11-beta-hydroxylase deficiency
	GAA	17q25.3	202300	AR	Glycogen storage disease II
	GLA	Xq22.1	300644	XL	Fabry disease; Fabry disease, cardiac variant
	HSD11B2	16q22.1	218030	AR	Apparent mineralocorticoid excess
	ITM2B	13q14.2	603904	AD	Retinal dystrophy with inner retinal dysfunction and ganglion cell abnormalities; Dementia
	MMACHC	1p34.1	609831	AR	Methylmalonic aciduria and homocystinuria, cblC type
	MTHFR	1p36.22	607093	AR	Homocystinuria due to MTHFR deficiency
	MMUT	6p12.3	609058	AR	Methylmalonic aciduria, mut (0) type
	PDE3A	12p12.2	112410	AD	Hypertension and brachydactyly syndrome
	PRKG1	10q11.2-q21.1	176894	AD	Aortic aneurysm, familial thoracic 8
	SAMHD1	20q11.23	612952	AR	Aicardi-Goutieres syndrome 5
	SCNN1B	16p12.2	600760	AD	Bronchiectasis with or without elevated sweat chloride 1
	SCNN1G	16p12.2	618114	AD	Liddle syndrome 2

**Table 2:** Summary of the genetic test results of 65 probands analyzed. CRGVs, clinically relevant genetic variants; VUSs, variants of unknown significance.

	High Probability Group, HPG (*n* = 22)			Low Probability Group, LPG (*n* = 43)		
		Gene	Variant		Gene	Variant
**CRGVs**						
No. of cases, %	4 (18.2)	ADA2	NM_001282225.2:exon2:c.G139A:p.G47R in homozygous state	0 (0.0)		
		NOTCH3	NM_000435:c.C619T:p.R207C in heterozygous state			
		ABCC6	NM_001171.6:c.C3421T:p.R1141Ter in heterozygous state			
		APP	NM_000484.4:c.2077G>A:p.Glu693Lys in heterozygous state			
**VUSs**						
No. of cases, %	5 (22.7)	GAA	NM_000152:c.G2700T:p.Q900H in heterozygous state	2 (4.6)	NOTCH3	NM_000435.3:c.1774C>A:p.Arg592Ser in heterozygous state
		NOTCH3	NM_000435:c.G3893T:p.C1298F in heterozygous state		COL4A2	NM_001846:c.G941A:p.G314E in heterozygous state
			NM_000435:c.C1662G:p.C554W in heterozygous state			
		GLA	NM_000169:c.G427A:p.A143T in homozygous state			
		HSD	NM_000196:c.T770C:p.V257A in heterozygous state			

## Data Availability

The data supporting the findings of this study are available from the corresponding author upon reasonable request.

## Supplementary Materials

The following supporting information can be downloaded at: https://www.mdpi.com/article/10.3390/genes17010098/s1, Table S1: Demographic, clinical, and radiological characteristics of the study group.

## Abbreviations

The following abbreviations are used in this manuscript:

cSVD	cerebral small vessel disease
hcSVD	hereditary cerebral small vessel disease
NGS	next-generation sequencing
WES	whole-exome sequencing
WGS	whole-genome sequencing
TOAST	trial of org 10,172 in acute stroke treatment
CSS	causative classification system for ischemic stroke
WMHs	white matter hyperintensities
PVSs	peri-vascular spaces
CMBs	cerebral microbleeds
ICH	intracerebral hemorrhage
BMI	body mass index
HPG	high-probability group
LPG	low-probability group
ACMG	american college of medical genetics and genomics
CRGVs	clinically relevant genetic variants
VUSs	variants of unknown significant
DADA2	adenosine deaminase 2 deficiency
CADASIL	cerebral arteriopathy with subcortical infarcts and leukoencephalopathy
